# Digging the optimum pit: antlions, spirals and spontaneous stratification

**DOI:** 10.1098/rspb.2019.0365

**Published:** 2019-03-27

**Authors:** Nigel R. Franks, Alan Worley, Max Falkenberg, Ana B. Sendova-Franks, Kim Christensen

**Affiliations:** 1 School of Biological Sciences, University of Bristol, 24 Tyndall Avenue, Bristol BS8 1TQ, UK; 2 Blackett Laboratory, Imperial College London, South Kensington Campus, London SW7 2AZ, UK; 3 Centre for Complexity Science, Imperial College London, South Kensington Campus, London SW7 2AZ, UK; 4 Department of Engineering Design and Mathematics, UWE Bristol, Frenchay Campus, Coldharbour Lane, Bristol BS16 1QY, UK

**Keywords:** animal traps, spontaneous stratification, granular materials, optimized construction, self-organization, extended phenotype

## Abstract

Most animal traps are constructed from self-secreted silk, so antlions are rare among trap builders because they use only materials found in the environment. We show how antlions exploit the properties of the substrate to produce very effective structures in the minimum amount of time. Our modelling demonstrates how antlions: (i) exploit self-stratification in granular media differentially to expose deleterious large grains at the bottom of the construction trench where they can be ejected preferentially, and (ii) minimize completion time by spiral rather than central digging. Both phenomena are confirmed by our experiments. Spiral digging saves time because it enables the antlion to eject material initially from the periphery of the pit where it is less likely to topple back into the centre. As a result, antlions can produce their pits—lined almost exclusively with small slippery grains to maximize powerful avalanches and hence prey capture—much more quickly than if they simply dig at the pit's centre. Our demonstration, for the first time to our knowledge, of an animal using self-stratification in granular media exemplifies the sophistication of extended phenotypes even if they are only formed from material found in the animal's environment.

## 1. Introduction

The extended phenotype concept pioneered by Dawkins [1] emphasizes the evolutionary importance of structures beyond the body of the organism. These include, most obviously, nests and tools [2]. True extended phenotypes are vital to those that deploy them [3]. In this light, the nests, traps and burrows that animals build may be much more important than tools because the former are often used every day, whereas tools are typically rarely employed even by the few animals that use them [4]. Thus, chimpanzees build treetop nests every night (and sometimes during the day) to ensure their safety but they use tools in less than 1% of their feeding activity over the course of a year (R.W. Wrangham, cited within [4]). Hence, we share the viewpoint of Hansell & Ruxton [4] that the buildings that many animals make (such as nests, prey-traps and mate-attracting structures, e.g. bird bowers and mole-cricket burrows) deserve at least as much attention as tools. Tool-use by animals attracts disproportionate attention because it is assumed that it reveals new or special cognitive abilities [5,6]. However, is it more challenging for a bird to use a twig to spear an insect from a hole than to build a nest out of many twigs that can cradle eggs securely atop a tree in a gale? We think not. The sophisticated structures that animals build clearly warrant more attention; and those created by animals with small brains might caution against evoking higher cognition in the production of extended phenotypes in many other cases too.

**Figure 1.**
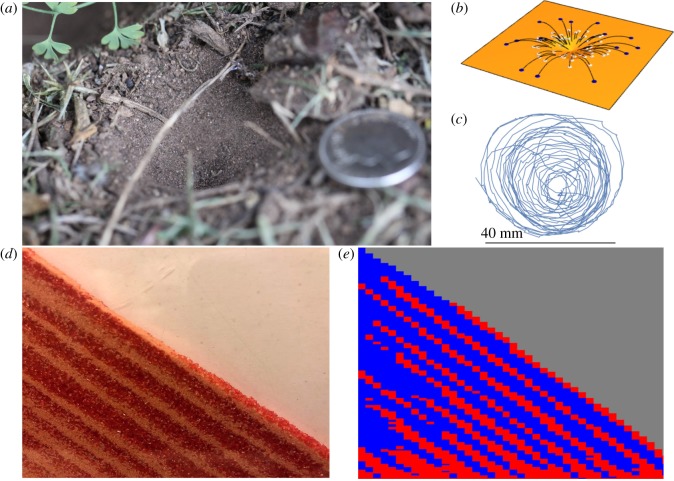
Antlion pits and spontaneous stratification. (*a*) An *Euroleon nostras* pit at the bottom of a hedge row in southwest Guernsey; coin diameter: 24 mm. (*b*) A cartoon of grain ejection and the segregation of ejected grains of two different sizes during pit construction: the larger (blue) grains are thrown on average further than the smaller (silver) grains in the same scoop of ejecta because their ratio of momentum to drag is higher. (*c*) A two-dimensional representation of the helical pit-construction trench; the irregular features are reversals of direction. (*d*) Close shot of an experimental ‘quasi-two-dimensional’, Hele-Shaw, cell as in [17]; a mixture of two grain types in equal volumes is poured from the top left corner and self-stratifies into successive layers of grains of each type [17]; the red rough sugar cubes are larger than the white round sand grains and have a greater angle of repose. (*e*) Simulated grains are poured as in a Hele-Shaw cell using the rules in our model; red: large grains; blue: small grains.

The construction of traps by animals is very sparsely distributed over the tree of life. For example, the only vertebrate species to construct animal traps is *Homo sapiens* [7]. Among invertebrate trap-builders, special materials seem essential and indeed, most animal trappers use silk. Orb-web spiders, in particular, deploy silk [8,9] to maximize benefits over costs [10–13]. Such is the case not only among the 10 000 web-building spider species, but also among 2000 species of net-building Trichoptera caddis-fly larvae and four species of *Archnocampa* gnat larvae*,* that use sticky silk traplines to capture prey [7]. Trap construction without silk is restricted to a few hundred antlion species and several wormlion species [14]. Antlions and wormlions construct ostensibly similar pits [15] even though pit-building antlions are Neuropterans and use spiral digging [14,16] (figure 1*c*; electronic supplementary material, video S1) while wormlions are Dipterans and use central digging [14,18]. Thus, antlions and wormlions are a remarkable example of partly convergent evolution. Their fascinating trap-building behaviour has been studied rigorously [14,19–29] and in particular by Jeffrey Lucas [19,23], whose seminal studies were the first to look formally at the biophysics of grain ejection by antlion larvae, including the segregation of ejected grains (figure 1*b*) [19]. Antlions get almost all the food they use, over their lifetime, via the pits they excavate: such pits are vital extended phenotypes and are made without any secreted material.

Phenotypic studies naturally examine issues such as development and structural efficiency and analogous issues should apply to extended phenotypes. Yet studies are rare that combine how extended phenotypes are constructed and how efficient they are. Such combined studies have mostly focused on the orb webs of spiders [2]. However, it might be argued that extended phenotypes based on a body's self-secretions (such as silk) are likely to be much more tightly under the influence of natural selection than extended phenotypes which use materials simply available in the environment. Hence, our goal here is to examine built structures made entirely from found materials.

A granular material, such as sand, is a collection of distinct particles that interact only by means of contact forces. Granular materials have fascinated scientists for centuries owing to their extraordinary properties. Sand may expand under shear as when wet sand dries around your feet while walking on the beach [30]. Oscillons and crystalline patterns [31] appear when a few layers of sand vibrate. Heap formation [32] and convection [33] occur when bulk sand is vibrated. Spontaneous self-organization is observed in mixtures of grains with different sizes. For example, the Brazil nut effect [34,35] occurs when particles segregate under vibration so that the larger heavier particles rise to the top against the gravitational gradient. By contrast, the phenomenon of spontaneous stratification is caused by avalanches, defined as the motion of grains that are linked in space and time [36,37], when large grains have a larger angle of repose than small grains and large and small grains form successive layers [17] (figure 1*d,e*).

Spontaneous stratification of granular materials has been examined by physicists [17] only since the late 1990's, well after Lucas's pioneering antlion study from the early 1980's [19], and thus, until now, it has not featured in antlion studies. Moreover, the efficiencies that might accrue from spiral digging have not been analysed. So, here we bring these two issues together and ask the questions: do antlions actively exploit the special properties of granular media and, if so, is their excavation optimized by spiral digging?

**Figure 2.**
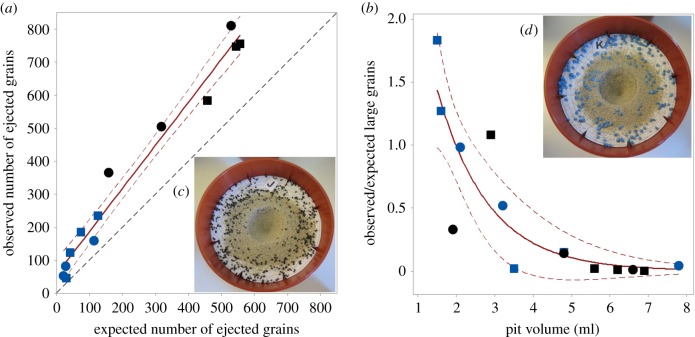
Experimental results. (*a*) Relationship between the observed number of ejected large grains and their expected number based on the substrate mixture and unbiased ejection; blue (black): 1–2 mm (1.5–3 mm) in diameter; dashed black line: line of equality; the *y*-difference between the regression line and the line of equality represents the number of observed large grains in excess of expected number. (*b*) Relationship between the ratio of the observed to the expected number of large grains (blue and black) in the pit wall and pit volume; circle (square): 20% (30%) volume fraction of large grains; solid red line: regression line, dashed red lines: 95% confidence interval for the regression line. Three and two of the 16 antlions were not included in (*a*) and (*b*), respectively, because they performed little or no pit building. (*c*–*d*) Experimental pot with a paper annulus over a mixture of silver sand and large black (blue) grains; the pit is in the middle of the hole in the paper annulus; small paper labels ‘J’ and ‘K’: pot IDs.

Antlions typically excavate their pitfall traps in sandy soils (figure 1*a*). We experimented upon antlions by giving them known mixtures of two different sizes of sand grains and a paper annulus to capture and analyse the sand-grain size ratios of the material they ejected from their pits (figure 2*c,d*). Our focus is on the ratios of ejected large grains relative to the original mixture rather than large debris that might be many times heavier than the antlion [38]. To assess the role of spiral digging, drag and redistribution of grains, we compare a spiral-digging model with three central-digging null models (table 1).

**Table 1.** The key predictions from the five models; ✓: present, ✗: absent, ✓*: partially present.

model variant	excess of large grains ejected	small-grain lining	pit completion time	large-grain excess at bottom of trench during construction	small grains at nadir
central digging, no redistribution	✗	✓*	✗	✗	✗
central digging, no drag	✓*	✓*	800	✗	✗
central digging with drag	✓*	✓	500	✗	✓*
spiral digging *r* = 25, with drag	✓	✓	300	✓	✓
spiral digging *r* = 50, with drag	✓	✓	600	✓	✓

## 2. Experimental material and methods

We collected 16 *Euroleon nostras* [39] antlion larvae from a field site in southwest Guernsey on 11 June 2016 and returned them unharmed to the same site on 16 June 2016. Intriguingly, these antlions reside at the bottom of hedgerows where their pits should be sheltered from rain. In other areas, antlions typically build in the open, say on mature stable sand dunes or at the edge of sandy paths in dry forests [40,41]. Hedgerow bottoms are full of debris and we reasoned that antlion larvae should have the ability to choose places for pit excavation which are relatively free of debris such as plant roots, fallen twigs and leaves. Indeed, antlion larvae do seem skilfully to choose sites where they have adequate space for their pits [42,43]. Hence, antlion larvae should centre their pits in suitable places provided for them.

We made up sand mixtures with three types of grain: natural, dry silver sand from a Guernsey beach, black silica grains (diameter: 1–2 mm, mean mass: 0.0078 g) and blue silica grains (diameter: 1.5–3 mm, mean mass: 0.028 g). The experiments used plant pots (top diameter: 14 cm, depth: 12 cm). Each was filled with a foundation layer of natural sand to a depth of 7 cm and then up to the height of the internal rim, 2.5 cm from the top, with one of four mixtures of large grains (black or blue) in natural sand at 20% or 30% by volume. On top of the sand, we placed a paper annulus (diameter: 12.6 cm) with a central hole (diameter: 4.0 cm).

Each of the 16 antlions was introduced to the centre of its pot (A to P, figure 2*c,d*) at 7.00 on 12 June 2016. We took the antlions' weights into account to produce a balanced design in their allocation to pots with black or blue large grains (electronic supplementary material, table S1). Many antlions began to construct pits within 1 h. Final photographs of the plant pots were taken at 4.30 on 14 June 2016. All the ejected sand on the paper was then recovered with an aspirator and sieved to separate the coarse coloured sand (black or blue) from the silver beach sand. Both components were weighed. The total numbers of coloured sand grains ejected were calculated from these weights after weighing 50 sand grains of each colour as reference samples.

The photographs were used to record the final location of each coloured sand grain. This was done by hand and eye using the mouse-based sequential numbering procedure in ImageJ software [44]. This labour-intensive method was preferred over automated methods for its greater accuracy. Such photographs were also used to count the numbers of coloured grains that were visible in the conical pit walls at the end of the process (electronic supplementary material, figure S2).

All experimental data analyses were carried out in Minitab 17 and 18 [45].

## 3. Experimental results

(i) The antlions preferentially ejected larger grains (figure 2*a*). Large grains (both blue and black) were ejected 1.30 times, 95% confidence interval (CI) (1.15, 1.45), more often than expected from the numbers that should have been present given the ratio of grain sizes in the initial substrate mixture and unbiased ejection (linear regression: intercept = 57.7, 95% CI (11.4, 104.1), *R*^2^ = 97.1%, Normality test for residuals, Anderson-Darlin test statistic (AD) = 0.239, *n* = 13, *p* = 0.723).
(ii) The larger the pit, the rarer visible large grains were in its conical walls (nonlinear regression model of exponential decay: constant = 4.4, 95% CI (1.9, 28.2), exponent = −0.75, 95% CI (−1.86, −0.37); figure 2*b*). This confirms the results from earlier studies [14,19] that completed pits are preferentially lined with fine sand grains (figures 2*c,d*). For a few pit volume values, the large grains in the pit wall were more than expected (figure 2*b*). This could have happened in part because smaller pits may not facilitate sufficient stratification [46]. Our finding that larger avalanches occurred as antlions increased their pit volumes (electronic supplementary material, figure S1) is also consistent with the preferential fine-sand lining of completed pits since fine grains have a smaller angle of repose and avalanches are more likely [17].
(iii) The average ratio of initial spiral radius to pit radius was 0.525, 95% CI (0.456, 0.618). This is the reciprocal of the slope for the linear regression through the origin for the relationship between initial spiral diameter and pit diameter (electronic supplementary material, figure S3, linear regression, *R*^2^ = 96.7%, Normality test for residuals, AD = 0.241, *n* = 9, *p* = 0.686). This 95% CI overlaps with the range 0.54–0.73 reported previously [16] and also includes the 0.60 ratio from the spiral-digging model (see later).
(iv) The final pit radius ranged between 12 and 23 mm (mean = 17.5 mm, median = 17.8 mm, s.e. = 0.98, *n* = 14; electronic supplementary material, table S1, col. 5).

## 4. Modelling methods

An antlion digging a conical pit is a complex phenomenon. To gain insight into the pertinent processes and the reasons antlions employ spiral digging, we formulate a computational model with the most essential features.

We take inspiration from classical work on self-organization in granular media [17,36,37,47–49]. Consider a granular mixture of small and large grains on a one-dimensional lattice with *L* sites *i* = 1*,*2, … , *L*, representing a cross-section through a real experimental pit (electronic supplementary material, figure S4*c*). Small and large grains have heights and volumes of 1 and 2, respectively. The height at site *i*, *h_i_*, is calculated as the sum of small and large grains at the site where the local slopes at each site are *z_i_*^Left^ = *h_i_* −*h_i_*_−1,_ and *z_i_*^Right^ = *h_i_* −*h_i_*_+1_.

An avalanche will occur in the granular medium if the local slope exceeds a threshold. Large grains can accommodate a steeper slope than small grains and small grains sitting on large grains are more stable than large grains sitting on small grains (electronic supplementary material, figure S4*a*). This rule is responsible for stratification in the model. A grain topples to the left, or right, if the local slope to the left, or right, exceeds a critical slope, *z_i_^c^*. If both *z_i_*^Left^ and *z_i_*^Right^ exceed the critical slope, the grain topples in the direction of the steepest slope or randomly to one of the sides if *z_i_*^Left^ = *z_i_*^Right^ > *z_i_^c^*.

We define the avalanche size as the total number of topplings in a pit at time *t*, weighted by the grain size, i.e. a large (small) grain toppling contributes 2 (1) units to the avalanche size. This ensures that the avalanche size corresponds to the total volume through which material topples.

In the initial state, small and large grains are added randomly to each site until *h_i_* *=* *H or H* + 1 (electronic supplementary material, figure S4*d*) with probabilities such that large grains occupy 25% of the grain mixture's volume (the midpoint of the 20 or 30% experimental volumes). We define a removal window of dimensions 5 × 5 (width × depth) as the material an antlion throws at each time step in the digging process (electronic supplementary material, figure S4*b*). The removal window is centred at a given lattice site which can be moved to mimic the antlion's spiral motion (electronic supplementary material, figure S4*c*). Grains may topple into the void until stability is reached. Applying a simple Stokes' drag approximation, Newton's second law determines the trajectory of thrown grains according to:

4.1 $$\begin{array}{rl} \frac{dv_{x}}{dt} & =-\alpha v_{x},\;\;\\ \;\frac{dv_{y}}{dt} & =\;-\alpha v_{y}-g, \end{array}$$

where *v_x_* and *v_y_* are the horizontal and vertical components of the grain velocity, respectively, *g* is the gravitational acceleration and $\;\alpha =g/v_{T}$ is the drag coefficient where *v*_T_ is the grain's terminal speed of 150 cm s^−1^ and 1000 cm s^−1^, for small and large grains, respectively, based on experimental results [50]. Hence, the drag coefficient, *α*, is larger for small grains than for large grains. Grains are thrown with an initial speed $v_{0}=(70+\;\delta v)\mathrm{cm}\;s^{-1}$ and direction $\theta_{0}={\left(50+\delta \theta \right)}^{\circ }$ (measured from a horizontal base line) with uniformly distributed noise δv ∈[−30,+30] and δθ ∈[−10,+10] based on experimental evidence [19]. To convert between trajectories with an associated real-world scale and the model, final trajectories are adjusted such that the pit generated from the model has approximate dimensions equivalent to those observed in our experiments.

Spiral digging can be implemented for a vertical cross-section across the pit by letting *i* *=* *L/2* denote the spiral centre and defining left and right boundary sites at a distance *r* from this centre (electronic supplementary material, figure S4*d*). We take a cut through the spiral and mark each intersection between the spiral and the cut with a black dot (electronic supplementary material, figure S4*c*). Following the spiral from the outside in, digging alternates between opposite sides of the cut from the centre, starting at an initial spiral radius *r*, and continues to move in until we reach the spiral centre (electronic supplementary material, figure S4*c*). We choose an initial radius of *r* = 25 and dig at each site four times—this ensures the spiral-digging removes the bulk of the material in the pit-creation process. The spiral reaches the centre after 8*r* time steps (electronic supplementary material, videos S2–S5). Pit completion is reached when the fraction of large grains in the removal window falls below a threshold (electronic supplementary material).

To assess the effect of spiral digging, we compared the spiral-digging model with the following three central-digging null models that are the limit of spiral digging when the initial spiral radius, *r* approaches 0: (i) central digging without redistribution: grains are removed permanently from the system and not redistributed; (ii) central digging without drag: grains are thrown using the same parameters as in the spiral-digging model but drag is neglected, *α* = 0, and hence, the average trajectories of small and large grains are identical; and (iii) central digging with drag: grains are thrown using the same parameters as in the spiral-digging model where we consider differences in the drag on small and large grains. This results in large grains being thrown further on average than small grains. We will omit the central digging without redistribution model from the figures.

Modelling results are largely robust against changes in drag implementation or removal window size (details in the electronic supplementary material). Pseudo-random numbers, used to assign grain sizes and critical slopes, and to choose the update-order of the sites, were generated with a Mersenne Twister routine to provide a high degree of statistical randomness and a long period.

## 5. Modelling results

### (a) Predictions from the spiral-digging model tested by the experimental data

**Figure 3.**
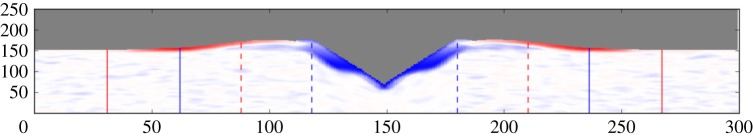
Image of the pit at *t* = 700 from the spiral-digging model with initial radius *r* = 25; an average result over 50 pits (see the electronic supplementary material, video S2 for an animation of the dynamics); red: excess of large grains, blue: excess of small grains, white: large and small grains mixed according to initial distribution (25% large grains by volume); solid vertical red (blue) lines: the maximum throwing distance of large (small) grains from the initial dig position at a spiral radius of *r* = 25 cells from the pit centre; dashed vertical red (blue) lines: the equivalent for large (small) grains thrown from the pit centre at pit completion.

**Figure 4.**
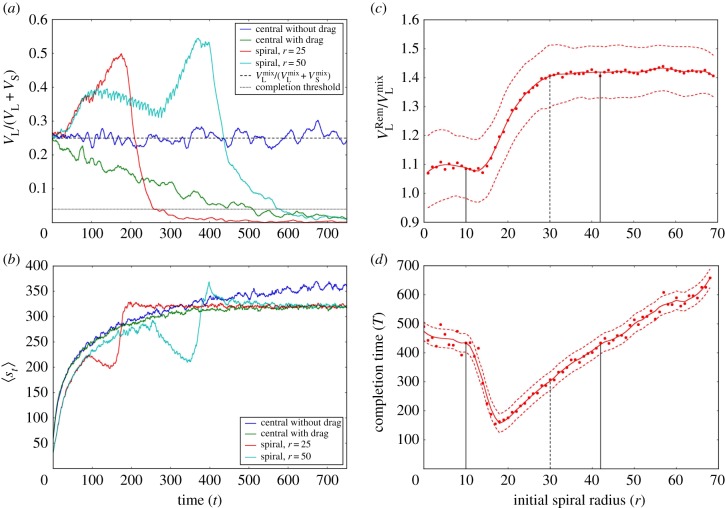
Model results. (*a*) The fraction of the removal window volume occupied by large grains and (*b*) the average avalanche size, 〈*s_t_*〉, over time for the spiral-digging and the null models with redistribution; solid lines: averages over 50 pit realizations; dashed line: expected volume fraction of large grains in the removal window based on large grains occupying 25% by volume in the original mixture; dotted line: 4% volume occupied by large grains in the removal window used to define a ‘completed pit’ (electronic supplementary material). (*c*) The ratio of the volume fraction of large grains ejected and large grains in the mixture, and (*d*) the time to pit completion against initial spiral radius, *r*, for spiral digging; red circles: averages over 50 pit realizations; solid red line: smoothed form of the relationship; dashed red lines: 95% CI envelope; dashed black line: final pit radius of 30 cells in the model (the average of 18 mm in the experimental pits, electronic supplementary material, table S1), at which the pit has a perfect small-grain lining; solid black lines: the spectrum of initial spiral radii, *r*, 10–42 (6–25 mm), where spiral digging offers substantial time savings over central digging. (Online version in colour.)

(i) At pit completion after spiral digging, the volume of large grains removed from the pit could be up to 1.40 times larger than expected from the initial volume of large grains in the mixture (figure 4*c*). This excess is within the 95% CI (1.15, 1.45) for the experimentally estimated population value (figure 2*a*). By contrast, central digging with and without drag shows only approximately 1.05 times excess of large grains removed (electronic supplementary material, figure S8*a*), which cannot account for our experimental results. This means that the antlions preferentially ejected larger grains in the proportions predicted by the spiral-digging model. The maximum excess is for a final pit radius of 30 cells, equivalent to the average of 18 mm final pit radius for the experimental pits (electronic supplementary material, table S1). There is a region of moderate radii where the fraction of large grains removed quickly increases before the result plateaus at large radii (figure 4*c*).
(ii) At pit completion after spiral digging, antlion pits are lined almost exclusively with small grains (figures 2*c*,*d* and 3; electronic supplementary material, S5*c*) and the profile of the pit is approximately constant with only small fluctuations (electronic supplementary material, videos S2–S5). Although pits are lined preferentially by small grains in all model variants owing to stratification (figure 3; electronic supplementary material, figure S5), the convergence to this result is fastest for spiral digging. For central digging, it is faster with drag than without drag or without redistribution. In the latter, a small number of large grains remains in the pit lining indefinitely (table 1).
(iii) The ratio between the initial radius, *r* ≈ 18, for which time to completion is minimized (figure 4*d*) and the final pit radius predicted by the model, *R* ≈ 30, is 0.60.
(iv) Pit completion (electronic supplementary material) takes about half the time for spiral digging with initial radius of 25 compared to the other models with redistribution (table 1). Yet, the final pits for a spiral of initial radius 25 or 50, and central digging with drag, all have similar dimensions: radius *R* ≈ 30 and depth *h* ≈ 100 or a two-dimensional volume ≈ 3000 cells, where a small (large) grain occupies 1 (2) cell(s). Hence, spiral digging with initial radius of 25 halves the time required to reach the equivalent final pit (figure 4*d*). In fact, the time savings afforded by spiral digging apply to initial spiral radii between 10 and 42 cells (6 to 25 mm). All experimental final pit radii fall within this range (electronic supplementary material, table S1), suggesting that antlions operate in the regime where spiral digging is highly effective with total time savings of up to 60% (figure 4*d*).

### (b) Hypotheses generated by the spiral-digging model

(i) The point after which further digging has a negligible effect on the pit profile (electronic supplementary material, videos S2–S5) predicts the pit depth at which the antlion should stop digging.
(ii) During pit construction, only spiral digging results in significant clustering of large grains at the bottom of the construction trench (figure 4*a* and table 1), and their efficient removal.
(iii) At pit completion, a build-up of small grains at the pit's nadir (the bottom of the pit) and large grains at its ramparts are present after spiral digging (figure 3; electronic supplementary material, figure S5*c* and S6) and, to some extent, after central digging with drag (table 1; electronic supplementary material, figure S5*b*).
(iv) Average avalanche size plateaus at a maximum (figure 4*b*) and the fraction of large grains in the removal window converges to zero when each model reaches pit completion (figure 4*a*, except central digging without drag). For spiral digging, avalanche size diminishes just before pit completion (figure 4*b*) when the antlion encounters large grains at high density in the pit centre (electronic supplementary material, videos S2–S5) and ejects them successfully. As no small grains can be removed near pit completion, immediately after large grains have been removed, the avalanche size reaches a maximum (figure 4*b*). This happens earlier for radius of 25 than 50 (figure 4*b*) because efficiency benefits are lost if the initial radius is too big (figure 4*d*). The antlion ejects material unnecessarily from regions with no influence on the final pit.
(v) Spiral digging yields time savings across a broad range of initial radii (figure 4*d*) because the rate at which the antlion encounters, and effectively ejects, large grains is increased (figure 4*a*) and the amount of ejected material that falls back into the pit is reduced since the initial radius is further away from the pit centre than in central digging. By contrast, during central digging with or without drag, large grains are not efficiently grouped and energy (and hence time) is lost because of ejected grains toppling back (as measured by the area between the curves for central and spiral digging, figure 4*b*). There is no time saving for spiral digging with initial radius of 50 (figure 4*d*) because the initial hole is unnecessarily large and fills up as the antlion approaches the spiral centre (electronic supplementary material, videos S4 and S5).
(vi) Spiral digging and stratification allow antlions to dig a deadly pit quickly. The avalanche size is a proxy for potential energy [51]. This reaches a maximum at pit completion. Hence, the final pit has a maximum potential-energy release per unit time.

## 6. Discussion

Using experimental tests in combination with modelling, we have shown, for the first time to our knowledge, that by constructing pits via a descending helical spiral trajectory [14,16,19] antlions exploit spontaneous stratification of the granular substrate [17] to produce a fine-grain slippery lining to their completed pit [14,16,19]. Furthermore, we demonstrate that spiral digging saves construction time compared to central digging for the following two main reasons. First, spontaneous stratification, resulting from avalanches initiated by the spiralling antlion stirring up the substrate, exposes the large grains at the bottom of the digging trench and results in their preferential removal as confirmed by our experimental results. Second, spiralling reduces the number of ejected grains returning to the pit because it enables the antlion to eject material initially from the periphery of the pit where it is less likely to topple back into the centre. Importantly, the time-savings afforded by spiral digging hold across a broad range of initial spiral radii offering the antlion significant flexibility in the pit construction process. Last, but not least, the conical pit resulting from the time-saving process of spiral digging maximizes avalanche potential energy and a ready supply of avalanche-promoting ammunition at its nadir. These large avalanches will carry potential prey, who step over the lip of the pit, swiftly and directly to the lethal jaws of the waiting antlion.

Our study indicates that only spiral digging can account for the key experimental results and that the spiral radius controls the total time the antlion needs to complete the pit. Spiral digging, however, is not inevitable. For example, wormlions do not excavate pits with a spiral trajectory but simply sit at the bottom of the pit throwing out grains [14,18] and they prefer very fine homogeneous substrates [52].

We have identified a potential rule of thumb for when an antlion should stop building its pit: ‘stop if only fine grains of sand are falling back into the pit’. At this point the pit would have become too deep to allow such grains to escape. This stopping rule would be robust to antlion size variability because it is likely to scale with concomitant differences in throwing distance.

Our study is unusual in that it combines an understanding both of how antlions make very effective pitfall traps and why the construction process saves time. By contrast, with the exception mainly of studies of orb web spinning spiders [2], most studies of animal constructions focus either on the how or the why. For example, the burrows of mole crickets are optimized to produce loud and pure songs, and they are possibly tuned by trial and error [53–55]. However, the actual method by which mole crickets sculpt key parts of their burrow, such as its exponential horn(s), is not fully understood (H.C. Bennet-Clark 2019, personal communication). Hence, much is understood about *why* the mole cricket burrow has a certain structure but much less is known about *how* it is tuned and constructed.

It may be much more straightforward for natural selection to optimize extended phenotypes that use self-secreted material than material that is simply available in the environment. The reason for this is that self-secreted material is likely to be under the direct influence of the organism's genes whereas selection cannot act on found material but only on the way it is used. Indeed, web-spinning spiders use self-secreted silk and for orb webs the way in which their arachnid builders optimally deploy frame silk and sticky (viscid) silk has been the subject of several beautiful studies [8,56]. For example, certain spiders can deploy double-stranded silk or single-stranded silk in ways that appropriately engineer the tensile strength and elasticity of their deadly traps [57].

Our study is, to our knowledge, the first to show animals making use of stratification in granular materials. Stratification is usually demonstrated with pouring as in a Hele-Shaw cell (figure 1*d*) [17] but during pit building, it results from the avalanches initiated by the antlion's digging. Moreover, we show that spiral digging combined with such stratification saves time compared to central digging. An antlion spending less time constructing its pit should be vulnerable for less time to its natural enemies (predators [25], parasitoids [58] and possibly even parasites ([14], pp. 139–141]). Our study is an example of the power of natural selection to produce extended phenotypes [1] even in small-brained animals. We hope that our results will stimulate further experimental studies and three-dimension modelling that considers the molecular forces between the grains [59] to address, among others, ultimate questions about energy efficiency and proximal mechanisms in terms of the behavioural rules of pit building, their flexibility and robustness to variation among antlions, the sizes of predator and prey [60], and substrate characteristics, including packing fraction and the effect of vibrations from the digging.

## Supplementary Material

Supplementary text

## Supplementary Material

Video S1

## Supplementary Material

Video S2

## Supplementary Material

Video S3

## Supplementary Material

Video S4

## Supplementary Material

Video S5

## Data accessibility

All experimental data are in the paper or electronic supplementary material. The program code for the models can be found at the Dryad Digital Repository: https://doi.org/10.5061/dryad.k7m5vf4 [61].
