# Supplementary material for: Digging the optimum pit: antlions, spirals and spontaneous stratification
Source: Proc Biol Sci. 2019 Mar 27;286(1899):20190365. doi: 10.1098/rspb.2019.0365 (PMC6452065; doi:10.1098/rspb.2019.0365)
Supplement: Supplementary text [file rspb20190365supp1.pdf]

Electronic Supplementary Material

Digging the Optimum Pit: Antlions, Spirals and Spontaneous Stratification

Nigel R. Franks[1]*, Alan Worley[1], Max Falkenberg[2,3], Ana B. Sendova-Franks[4], Kim Christensen[2,3]

[1]School of Biological Sciences, University of Bristol, 24 Tyndall Avenue, Bristol BS8 1TQ, UK.

[2]Blackett Laboratory, Imperial College London, South Kensington Campus, London SW7 2AZ, UK.

[3]Centre for Complexity Science, Imperial College London, South Kensington Campus, London SW7 2AZ, UK.

[4]Department of Engineering Design and Mathematics, UWE Bristol, Frenchay Campus, Coldharbour Lane, Bristol BS16 1QY, UK.

*Corresponding author. Email: nigel.franks@bristol.ac.uk

This file includes:

- Captions for:
  - Video S1. Time-lapse recording of pit construction by an *Euroleon nostras* antlion.
  - Video S2. Average pit development for a spiral of initial radius $r = 25$.
  - Video S3. Single pit development for a spiral of initial radius $r = 25$.
  - Video S4. Average pit development for a spiral of initial radius $r = 50$.
  - Video S5. Single pit development for a spiral of initial radius $r = 50$.

- Supplementary text in:
  - Section S1. Additional experimental methods and results.
  - Section S2. Additional model methods and results.

- Figure S1. Estimated volume of sand ejected versus pit volume.
- Figure S2. Counting by eye is representative.
- Figure S3. Linear regression through the origin for the empirical relationship between pit diameter and initial spiral diameter.
- Figure S4. Illustrations of key spiral-digging model details.
- Figure S5. A sideview image of the pit at t=800 (to get the last remaining large grains out the pit centre) from the other three null models with redistribution.
- Figure S6. A sideview image of the pit at t=700 from a single iteration of the spiral-digging model with initial radius of 25 and 50.
- Figure S7. A sideview image of the pit at t=700 from spiral digging of initial radius of 25 for two additional sizes of the removal window.
- Figure S8. The excess ratio of large grains removed from the pit in the four model variants with redistribution (Table 1) and the excess ratio of large grains in the removal window of the spiral-digging model with small initial radius.
- Figure S9: The time to pit completion against initial spiral radius, *r* for spiral digging when the completion threshold is changed from 4 to 5% to be compared with figure 4*d*.
- Figure S10: The ratio of the volume fraction of large grains ejected and large grains in the mixture against initial spiral radius, r for spiral digging based on large grains occupying (a) 20%, (b) 25% (as in figure 4*c*) and (c) 30% by volume in the original mixture.
- Table S1. Characteristics of the antlions and their pits from the 16 experimental replicates.

Video S1. Time-lapse recording of pit construction by an *Euroleon nostras* antlion. The pit was constructed in a tub of size 120x120x80mm filled with beach sand and with a constraining circular acetate ring with a 100mm diameter. Still images were recorded at 0.5fps with a Canon 5D MK II camera. The behaviour of the antlion over 25min was converted into 26s of time-lapse video at 29fps. Note there is a reversal in the direction of spiral digging at 4s from the start, a short period of digging at the bottom of the pit between 23 and 26s and also that the throwing range declines with time as the pit gets deeper.

Video S2. Average pit development for a spiral of initial radius $r = 25$. The animation is for the model with large grains occupying 25% of the mixture's volume. The evolution is averaged over 50 pits. The image has been smoothed using a 5×5 square discrete convolution; red: excess of large grains, blue: excess of small grains, white: large and small grains mixed according to the original distribution.

Video S3. Single pit development for a spiral of initial radius $r = 25$. The animation is for the model with large grains occupying 25% of the mixture's volume. The dynamics are for a single pit. At completion, the pit is lined by small grains with a pool of small grains at its centre. The pit wall is dominated by small grains sitting on a base of large grains. See video S5 for stratification ridges. Large grains have been removed preferentially and line the ramparts; red: large grains, blue: small grains.

Video S4. Average pit development for a spiral of initial radius $r = 50$. The animation is for the model with large grains occupying 25% of the mixture's volume. The evolution is averaged over 50 pits. The breadth of the area where the large grain density has been changed from the initial distribution indicates that these pits are not efficient – large grains have been removed from a significant fraction of the substrate lying outside the final pit radius of approximately 30 cells. These regions have no influence on the form of the final pit and yet significant time has been wasted digging through these regions and ejecting large grains. Material has been filtered which does not contribute to the final pit profile; red: excess of large grains, blue: excess of small grains, white: large and small grains mixed according to the original distribution.

Video S5. Single pit development for a spiral of initial radius $r = 50$. The animation is for the model with large grains occupying 25% of the mixture's volume. The dynamics are for a single pit. At completion, the pit is lined by small grains with a pool of small grains at its centre. The wall of the pit is dominated by small grains sitting on a base of large grains. There is evidence of the self-stratification process that facilitates the preferential removal of large grains by the antlion during pit construction, as indicated by the presence of large grain ridges in the pit walls (figure 1*d*-*e*). Once ejected, the large grains line the ramparts; red: large grains, blue: small grains.

Section S1. Additional experimental methods and results.

Additional details for the general experimental methods. The three types of sand used were (a) natural Guernsey silver sand (approximately 75% of these grains had minimum diameters between 0.250 and 0.425mm); (b) black silica grains (crystal-quartz-gravel, diameter: 1-2mm, mean grain mass = 0.0078g, with rounded edges, www.dennerle.com) and (c) blue silica grains (diameter: 1.5-3mm, mean grain mass = 0.028g, resin coated, Pettex Mediterranean Blue, www.pettex.co.uk). The mass of each of the three types of sand grain was a fraction of the antlion average mass (mean=0.08g, median=0.07g, Table S1).

In each plant pot (12cm high with a diameter of 14cm at the top and 9.5cm at the base; Plantpak 13F/14A), we inserted a flower pot saucer (Ward Darlaston, 10cm, Wednesbury West Midlands) to prevent the sand from escaping. The plant pots had an internal rim 2.5cm from the top with 8 projections. These projections secured the position of the paper annulus when the plant pot was filled with sand up to its internal rim (figure 2*c*-*d*).

The four variants of sand mixture were prepared in large plastic tubs using two types of small plastic beaker, one that held 86ml and one that held 27ml of sand when full and levelled-off. Four 86ml measures of fine sand were added to the large tub to which either three or five 27ml measures of either black or blue sand were also added. This yielded either a 20 or 30% composition of the large grains (either black or blue) by total volume (Table S1, col. 3). Homogeneity of the mixture was achieved via gentle stirring with a plastic teaspoon. Care was taken to stir the mixture and not to shake it to avoid grain separation through the Brazil nut effect [32–34]. After adding the foundation layer of silver sand to a depth of 7cm, the mixture was carefully transferred to the plant pot with a spoon and levelled without excessive pressure.

To steer the antlions to build their pits near the centres of the pots, we placed the paper annulus with its central hole gently atop the sand in each pot (figure 2*c*-*d*). Each paper annulus was inscribed with an "N" to represent geographic North and orientated accordingly. The paper served later to separate out and identify all the ejected material.

Additional details for the experimental data analysis. The analysis in figure 2*a* is a linear regression of the observed number of large grains ejected against the expected number of large grains ejected (cols. 13-14 Table S1).

The expected number of large grains visible in the pit wall for the non-linear regression model in figure 2*b*, was calculated with reference to the concentration of large grains in the original mixture (Section S1, Analysis of large grains visible in the pit wall). The ratio of observed to expected large grains in the pit wall (col. 20, Table S1) was calculated by dividing the values in col. 19 by those in col. 17. Pit volume values were taken from col. 9 (Table S1).

We were justified in fitting a linear regression model through the origin for the empirical relationship between pit diameter and initial diameter (figure S3; Section S1, Spiral pit-building) because the intercept was not significantly different from 0 (Linear regression: intercept=9.62, 95% CI (-3.81, 23.05), gradient=1.45, 95% CI (0.77, 2.14), R^2=78.3%, Normality test for residuals, AD=0.260, N=9, p=0.617).

Test of reliability for grain-counting method. The paper annulus method reveals which grains have been ejected (Table S1). Individual grains can be recorded from photographs. Some grains, however, might be hidden behind others but this seems not to cause biases as the numbers of coloured grains counted from the photographs correlates very strongly indeed with the numbers of coloured grains retrieved from the paper annulus by aspiration, separated by sieving and numerically estimated by weighing (figure S2).

Spiral pit-building. The data for figures 1*c* & S3, were produced by introducing an antlion into a container 120x120x80mm deep filled to a depth of 45mm with sieved, dry beach sand. We photographed the containers from above with a Canon 5D MK II camera and a Canon EF 40mm f/2.8 STM lens triggered by an external timer to take full-frame still photographs at 2s intervals. The runs typically lasted ~5h/18000s/9000 images. To form the track image shown in figure 1*c*, we extracted the x-y coordinates of an antlion from every image in which it was visible. Using the mouse position beginning at the first time at which it made a clear circle, we recombined all extracted x-y coordinates to reconstruct the antlion's spiral track. In this example, the antlion made 26 circuits, taking 2340s to complete the pit, during which it made four changes of direction. The analysis in figure S3 is based on the tracks of nine antlions introduced into containers set up in the same way.

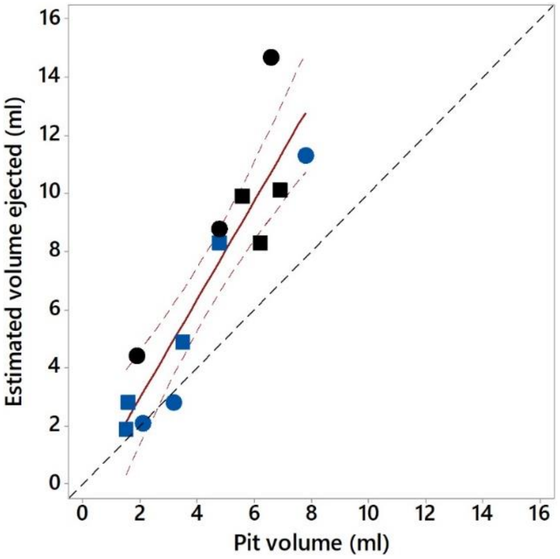

Figure S1. Estimated volume of sand ejected versus pit volume. There is a difference between the estimated pit volume and the estimated volume of sand ejected in the 13 experimental replicates. This may be explained by a combination of factors: larger avalanches as the antlion attempts to increase the pit size and/or the ground sinking, resulting in an underestimate of pit depth or an effect of the packing fraction of the ejected material. Linear regression: intercept=-0.4, 95% CI (-2.9, 2.0), gradient=1.69, 95% CI (1.19, 2.20), R^2=83.06%, Normality test for residuals, AD=0.318, N=13, p=0.497; blue and black symbols correspond to pits with blue and black large grains, respectively; circle: 20% volume fraction of large grains; square: 30% volume fraction of large grains; solid red line: regression line, dashed red lines: 95% CI for the regression line; dashed black line: unity line. Three of the 16 antlions were not included because they performed little or no pit building (Table S1, cols. 9 and 15).

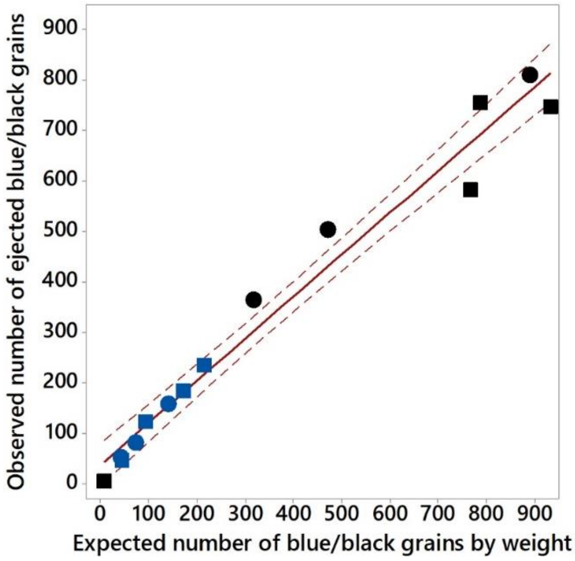

Figure S2. Counting by eye is representative. Extremely strong correlation between visible (blue or black) grains in the photographs and the numbers revealed by weighing. This suggests that the analyses on the visible grains in the photographs should not introduce significant biases. Linear regression: intercept=38.1, 95% CI (-5.0, 81.2), gradient=0.83, 95% CI (0.74, 0.92), R^2=97.21%, Normality test for residuals, AD=0.204, N=14, p=0.842; circle: 20% volume fraction of large grains; square: 30% volume fraction of large grains; solid red line: regression line, dashed red lines: 95% CI for the regression line. Two of the 16 antlions were not included because they did not make pits (Table S1, col. 10 divided by mean grain mass, black: 0.0078g, blue: 0.028g, and col. 13).

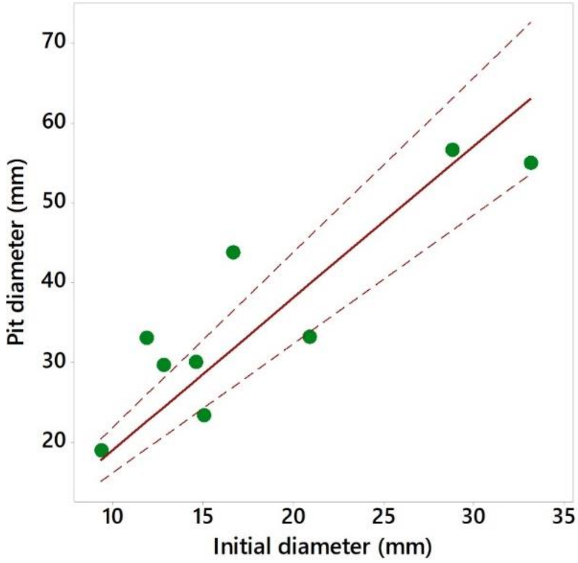

Figure S3. Linear regression through the origin for the empirical relationship between pit diameter and initial spiral diameter for nine antlion larvae (see main text and ESM, Spiral pit-building for more details); solid red line: regression line, dashed red lines: 95% CI for the regression line.

Analysis of large grains visible in the pit wall. We compared the observed number of large grains visible in the pit walls with the number expected based on their concentration in the original mixture. The theoretical calculation uses spheres representing the large grains placed randomly, but without overlap and at the appropriate number density, in a cube. The area of the cube face is set equal to the measured pit surface area. Using repeated random choices of the sphere placement, and by counting the number of spheres intersected by random horizontal slices taken through the cube, we obtain a measure of the mean expected number of grains visible in the pit wall and its variance (Table S1, cols. 17-18).

The pit depth *h* and radius *R* are used to calculate the surface area of each pit, $A = \pi R(R^2 + h^2)^{1/2}$, and the four grain densities (Table S1, col. 16) are derived from direct measurements on our mixtures. For the calculation of the surface density of visible grains, the mean grain diameters for the black and blue gains were estimated as *d* = 1.778mm and *d* = 2.72mm, respectively, and the adopted cube side $k = A^{1/2}$ was taken in each case to match the area of the pit. For each of the four grain-type (grain-concentration) choices we tested 100 different sphere configurations and took 10 equally-spaced slices through each. A grain was counted as intersecting a slice at height *m* if the *z*-coordinate of its centre satisfies $abs(m - z) < s$, where *s* is the distance at which the large grain appears as a circle of area 1.0mm^2 after removing all material above *m* and cutting through any grain intersected by the slice. Mean grain counts and standard deviations are based on 1000 slices (Table S1, cols. 17-18).

1	2	3	4	5	6	7	8	9	10	11	12	13	14	15	16	17	18	19	20
**Large grain type**	**Ant-lion/ pot**	**Vol. frac. of large grains**	**Ant-lion wt (g)**	**Pit diam., 2*R (mm)**	**Pit depth, h (mm)**	**Pit surf. area (mm^2)**	**Angle of pit wall (deg)**	**Pit vol. (ml)**	**Black or blue ejecta wt (g)**	**Silver grains ejecta wt (g)**	**Total ejecta wt (g)**	**No. of large grains in ejecta (obs)**	**No. of large grains in ejecta (expc)**	**Estim-ated vol. eject-ed (ml)**	**No. of large grains per ml of mixt.**	**Pred. no. of large grains in pit wall**	**s.d. of pred. no. of large grains in pit wall**	**No. of vis. large grains in pit wall**	**Vis./ pred. large grains in pit wall**
Blue	A	20%	<0.01	27	11	750	39	2.1	1.18	1.82	3.00	53	21	2.1	10	18.3	2.7	18	0.98
Black	B	20%	0.03	44	13	1770	31	6.6	6.94	13.66	20.60	811	529	14.7	36	86.8	7.4	1	0.01
Blue	C	30%	0.04	25	10	630	39	1.6	2.64	1.53	4.17	123	42	2.8	15	22.9	3.2	29	1.27
Black	D	30%	0.04	42	15	1710	36	6.9	6.15	8.60	14.75	756	556	10.1	55	128.0	7.8	0	0.00
Blue	E	20%	0.07	32	12	1020	37	3.2	2.01	2.07	4.08	82	28	2.8	10	24.9	3.8	13	0.52
Black	F	20%	0.06	26	11	690	40	1.9	2.48	3.68	6.16	365	158	4.4	36	33.6	4.8	11	0.33
Blue	G	30%	0.05	35	11	1140	32	3.5	4.79	2.49	7.28	185	74	4.9	15	41.4	4.4	1	0.02
Black	H	30%	0.04	39	14	1470	36	5.6	7.28	7.12	14.40	747	545	9.9	55	109.9	7.4	2	0.02
Blue	I	20%	0.07	46	14	1950	31	7.8	3.96	12.55	16.51	159	113	11.3	10	47.7	5.0	2	0.04
Black	J	20%	0.10	39	12	1410	32	4.8	3.68	8.55	12.23	505	317	8.8	36	69.6	6.5	10	0.14
Blue	K	30%	0.11	36	14	1290	38	4.8	6.03	6.32	12.35	236	125	8.3	15	47.0	4.7	7	0.15
Black	L	30%	0.11	41	14	1590	34	6.2	5.98	6.19	12.17	583	457	8.3	55	119.1	7.7	1	0.01
Blue	M	20%	0.19	*	*	*	*	*	*	*	*	*	*	*	10	*	*	*	*
Black	N	20%	0.13	*	*	*	*	*	*	*	*	*	*	*	36	*	*	*	*
Blue	O	30%	0.13	24	10	600	40	1.5	1.25	1.56	2.81	46	29	1.9	15	21.8	3.2	40	1.83
Black	P	30%	0.12	33	10	990	31	2.9	0.05	<0.01	0.06	6	0	0.0	55	73.8	6.3	80	1.08

Table S1. Characteristics of the antlions and their pits from the 16 experimental replicates. Col. 3 is the composition volume fraction of the large grains (blue or black) out of the total volume of grains when large grains and fine dry beach silver sand were initially added together in a mixing tub. The values in cols. 4-6, 10-11 are direct measurements made with a micro-balance or electronic digital callipers. Cols. 7-9 are calculated as $\pi R(R^2+h^2)^{1/2}$, $\tan^{-1}(h/R)$ and $\pi R^2 h/3$, respectively. Col. 12 is the sum of cols. 10 and 11, and the numbers in col. 13 are direct observations. Col. 14 was calculated as col. 15 times col. 16. Col. 15 is col. 12 divided by the grain density for the respective grain type (col. 1) and composition volume fraction of large grains (col. 3); blue, 20%: 1.460; black, 20%: 1.397; blue, 30%: 1.485; black, 30%: 1.459. For col. 16, the volume for a sample of the initial mixture times the respective volume fraction of large grains in col. 3 gives the volume of large grains and hence their weight when multiplied by the respective grain density (see explanation for col. 15 above). This weight of large grains in the mixture sample divided by the mean grain weight (black: 0.0078g, blue: 0.028g, see Methods) gives the number of large grains and hence the number per unit volume of mixture when divided by the volume of the initial sample. Cols. 17-18 were calculated according to the method in ‘Analysis of large grains visible in the pit wall’, Section S1. Col. 19 contains direct observations and col. 20 is the ratio of cols. 19 and 17. The pit characteristics for M and N are missing because these individuals did not build pits, and each ejected only 1 large grain. P built a very small pit and ejected 6 large grains. Hence, M and N were not included in any of the analyses, and P was included only in figures 2*b* & S2. Note that some of the pits were not perfectly lined with small grains because, as predicted by the model (figure 4*a*), just before pit completion, many large grains would be at its bottom.

Section S2. Additional model methods and results.

Model implementation. The model was implemented in *python2* using in-built lists and *numpy* arrays as the primary data structures.

At each time step, the number of small and large grains in the 5×5 removal window was recorded before ejection. After ejection, the total number of lattice sites moved by toppling grains was recorded. Each toppling was weighted by the grain size such that a small (large) grain toppling 1 site was considered an avalanche of size 1 (2). This ensures that the avalanche size is a proxy for the potential energy released. The recorded avalanche sizes are used to calculate pit statistics.

A typical simulation for spiral digging for 700 time-steps (which is approximately the maximum required to reach limiting pit behaviour) takes approximately 4 minutes on an Intel® Core™ i7-5600U CPU @ 2.6GHz processor. To ensure statistical results were of sufficient quality and precision, each simulation was repeated 50 times for each set of model parameters. Hence, for the completion-time curve shown in figure 4*d* in which 70 separate spiral radii were tested. This required a total simulation time of approximately 240h.

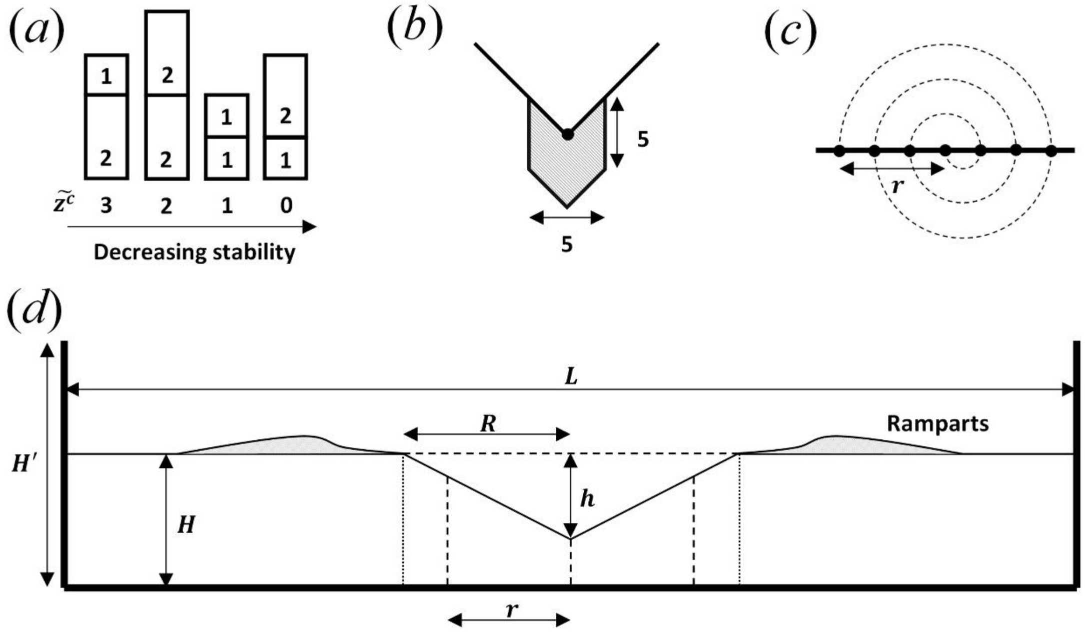

Figure S4. Illustrations of key spiral-digging model details. (*a*) The four possible stacks of small (1) and large (2) grains with their associated critical slopes, $\tilde{z}^c$, excluding the additional noise term, *δ*. (*b*) A depiction of the material included in the 5×5 (width × depth) removal window, centred at the digging location. (*c*) The assignment method of excavation sites in the model based on spiral digging in 2-D. (*d*) The principle dimensions of the model; solid black outline: walls at i = 0 and i = L + 1 for the pot containing sand; L: pot width; H: initial sand height; H': pot height; r: initial pit radius; R: final pit radius; h: final pit depth with the latter two measured from the initial sand height; ramparts: material that builds up at the edges of the pit with each throw.

Technical details for the spiral digging model. The heights at the boundaries $h_0 = h_{L+1} = H'$ (figure S4*d*) represent the walls of a square container initially filled with a randomized mixture of grains. The system size, *L,* is chosen such that grains never reach the boundaries. Introducing the notion of a 'grain stability', we assign heights of $h_S = 1$ and $h_L = 2$ and stabilities of $g_S = 1$ and $g_L = 2$ for small and large grains, respectively. The critical slope $z_i^c = 2g_i^B - g_i^T + \delta$ where g_i^T is the stability of the top grain, g_i^B the stability of the grain it is sitting on and $\delta \in \{2,3\}$ with equal probability. This ensures that large grains have a larger angle of repose than small grains and that small grains are more stable sitting on large grains than large grains

sitting on small grains. The inclusion of the δ term introduces a small amount of stochasticity into the model and ensures the critical slope is never smaller than 1, preventing grains from toppling unphysically from one site to another if the local slope is zero. Illustrations of the key model details and scales are shown in figure S4.

The effect of changing the size of the removal window. The volume of material an antlion throws at each time step does not have a large effect on the structure of pit digging. We tested for this effect by changing the size of the removal window from 5×5 to 3×3 and 15×15. In general, the qualitative results are the same (figure S7). The main quantitative differences are that the rate at which the final pit size is reached and the rate at which the pit becomes lined by small grains are both higher for larger removal windows. This is because, the granular layer active in any one time-step is thicker when the removal window is larger. Hence, stratification effects are stronger and can separate large and small grains more efficiently. The main noticeable difference in the pit profile is the thickness of the lining of small grains. This means that more large grains are removed for large removal windows because the wall lining is so much thicker. However, this is probably unrealistically large for an antlion. The size of the pool of small grains at the pit's nadir is also affected: a thicker pit lining is associated with a larger pool of small grains for spiral digging and central digging with drag.

Definition of a complete pit. We define pit completion as the time at which the fraction of the removal window occupied by large grains is less than 4% (figure 4*a*). This represents a single unit volume in a 5x5 removal window. Small changes to this threshold have only a small effect on the value of the initial spiral radius with maximum time savings (figures 4*d* & S9). As a principle, we find that at a certain point in the process of pit building in the spiral-digging model and the null models with redistribution, all thrown grains are small and they all fall back into the pit. At this stage the pit profile is approximately constant with small fluctuations - the antlion pit has reached a maximum size. This is determined by the angle of repose of the granular mixture and the throwing velocity. We refer to the pit at this stage as complete. Note that different antlions may be able to throw grains at different angles and at different velocities. Hence, this may result in variable pit dimensions between antlions. At pit completion, small grains cannot escape the pit, but large grains can still be ejected if present. Further digging would then be a Sisyphean task because small grains thrown from the bottom of the pit would always topple back down the pit walls to the antlion.

Pit volume at completion. For similar fractions of large grains in the original substrate and for a fixed throwing mechanism (i.e. grains are thrown with the same range of velocities and angles and the same drag parameters), pit volume is approximately constant. The final pit radius can be identified as the first initial spiral radius at which the large grain ejection fraction plateaus at a maximum. This is very close to 30 cells for 20, 25 and 30% fractions (figure S10). Hence, spiral digging and central digging with drag result in pits of approximately the same size. Central digging without drag results in slightly larger pits since the average distance a grain is thrown is larger. Central digging without redistribution does not result in a final pit size – the pit can grow indefinitely since there is no pit size above which grains cannot be removed.

Calculation of the excess of large grains removed from the pit. There is no unique definition for what is considered "out of the pit". As a first approximation, we assume any material above the height, H, of the original mixture (figure S4*d*) to be out of the pit. This in turn allows us to calculate the fraction of large grains in the removed volume and normalise this by the expected fraction of large grains in the original mixture (figures 4*c* & S8).

Dynamic spontaneous stratification. The spiral-digging model predicts that we will see little or no evidence of spontaneous stratification during the digging process (videos S2-S5)

because the antlion continuously removes preferentially the upper substrate layer, thereby effectively preventing the formation of the layers otherwise seen without removal.

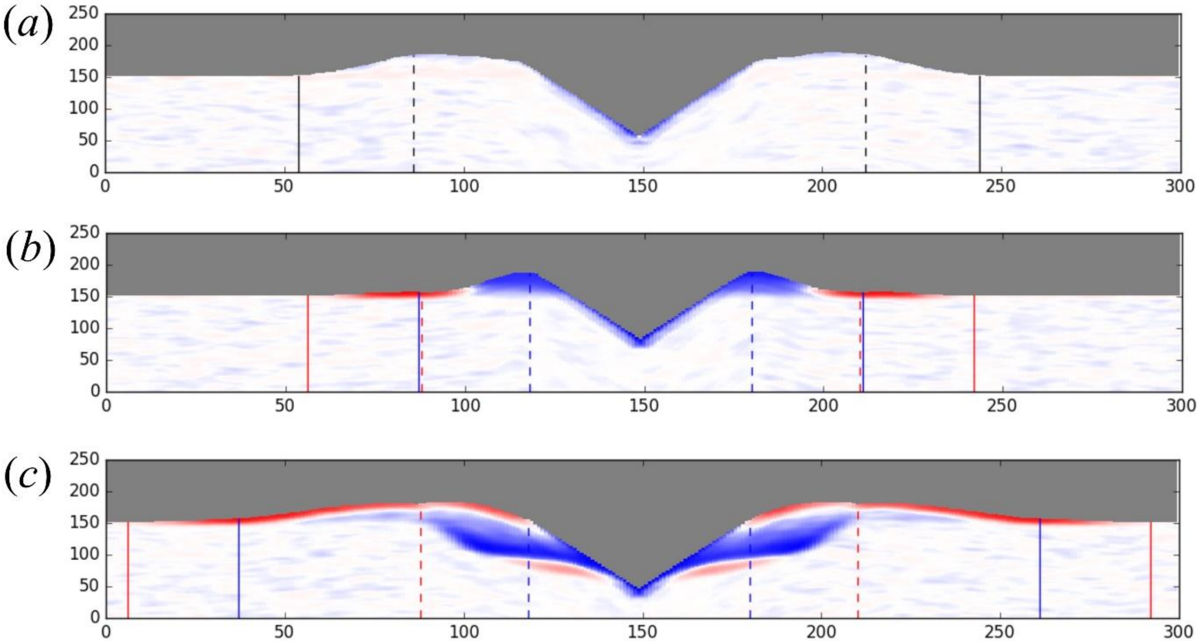

Figure S5. A sideview image of the pit at t=800 (to get the last remaining large grains out the pit centre) from the other three models with redistribution to complement figure 3: (*a*) central digging with no drag; it shows the role of stratification without other effects; (*b*) central digging with drag; (*c*) spiral digging of initial radius 50 (see video S4 for an animation of the dynamics). Each image represents an average result over 50 pits; red: excess of large grains, blue: excess of small grains, white: large and small grains mixed according to initial distribution (25% large grains by volume); solid red (blue) lines: the maximum throwing distance of large (small) grains from the initial dig position at a spiral radius of $r = 50$ cells from the pit centre for (*c*) and from the pit centre (r=0) before digging is initiated for (*b*); dashed red (blue) lines: the equivalent for large (small) grains thrown from the pit centre at pit completion; black solid (dashed) lines: the maximum throwing distance of both large and small grains when drag is neglected from the pit centre before digging is initiated (at pit completion).

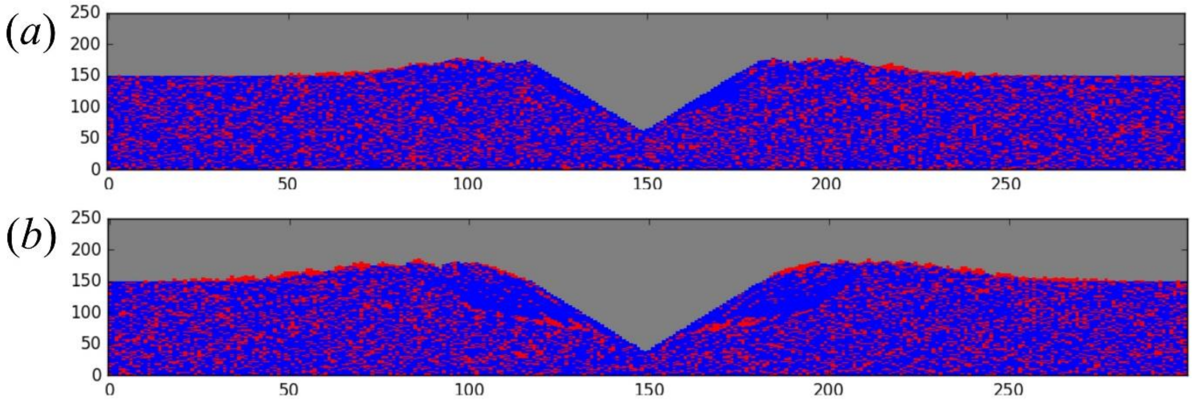

Figure S6. A sideview image of the pit at t=700 from a single iteration of the spiral-digging model with: (*a*) initial radius of 25 and (*b*) initial radius of 50 to complement figures 3 and S5; red: large grains, blue: small grains; results for different iterations could vary because of the stochastic elements in the model (e.g. the random distribution of large and small grains in the original mixture).

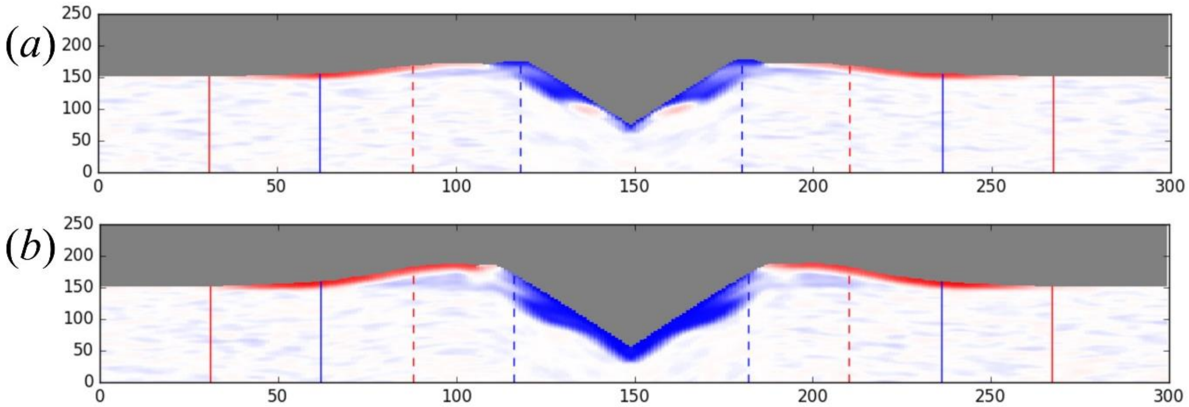

Figure S7. A sideview image of the pit at t=700 from spiral digging of initial radius of 25: (*a*) removal window of size 3×3 (width x depth); (*b*) removal window of 15×15 for comparison with figure 3, where the removal window is 5×5.

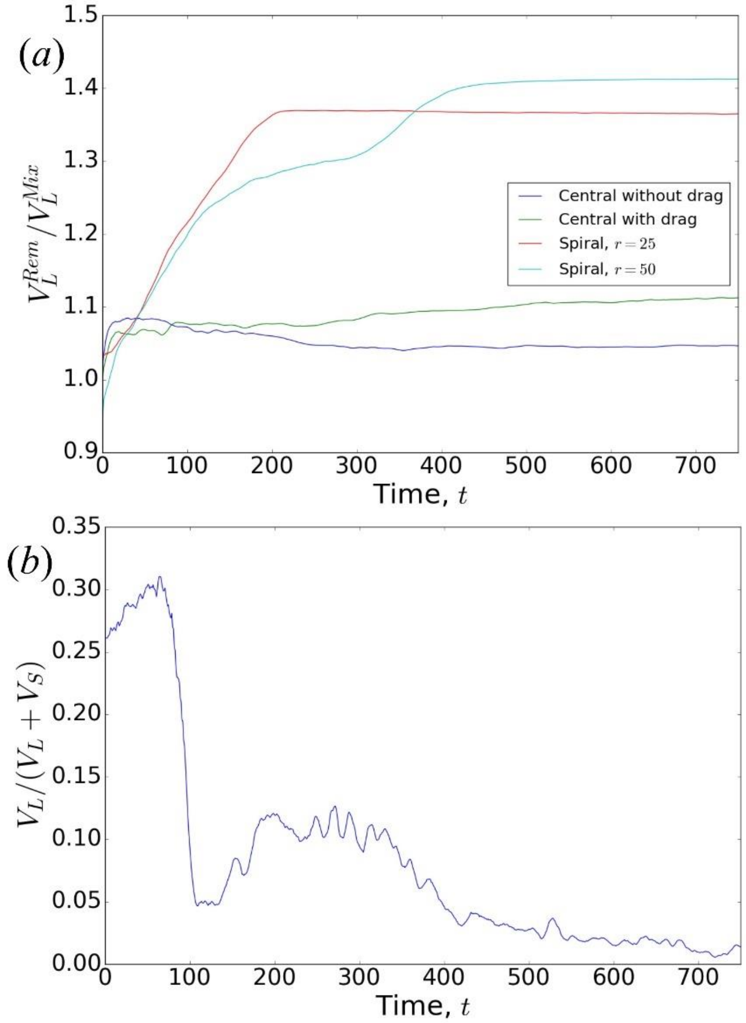

Figure S8. The excess ratio of large grains: (*a*) removed from the pit in the four model variants with redistribution over time (Table 1); each line represents 50 pit realisations with large grains occupying 25% of the original mixture's volume; (*b*) in the removal window of the spiral-digging model when the initial radius is too small, $r = 13$; such that the pit is not complete when the spiral reaches the centre; V_L^{Rem}: volume fraction of large grains removed from the pit during digging; V_L^{Mix}: volume of large grains in the original mixture; V_L: volume of large grains in the removal window; V_S: volume of small grains in the removal window.

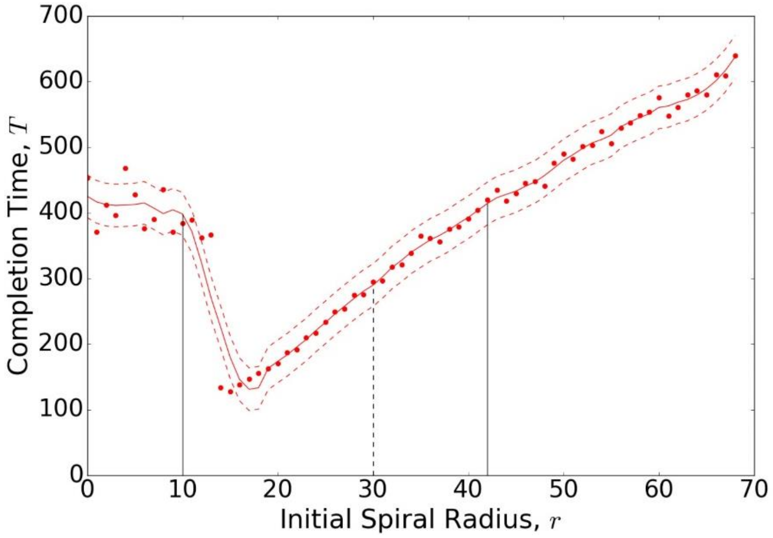

Figure S9. The time to pit completion against initial spiral radius, *r* for spiral digging when the completion threshold is changed from 4 to 5%, to be compared with figure 4*d*; red circles: averages over 50 pit realisations; solid red line: smoothed form of the relationship; dashed red lines: 95% CI envelope; dashed black line: final pit radius of 30 cells in the model (the average of 18mm in the experimental pits, Table S1), at which the pit has a perfect small-grain lining; solid black lines: the spectrum of initial spiral radii, r, 10-42 (6-25mm), where spiral digging offers substantial time savings over central digging. Changing the threshold from 4 to 5% makes little difference. Our aim is to find the time when there are no large grains left in the pit. Therefore, we want to set the threshold as low as possible. However, any lower than 4% and the time a pit crosses the completion threshold becomes undefined because of small amounts of noise.

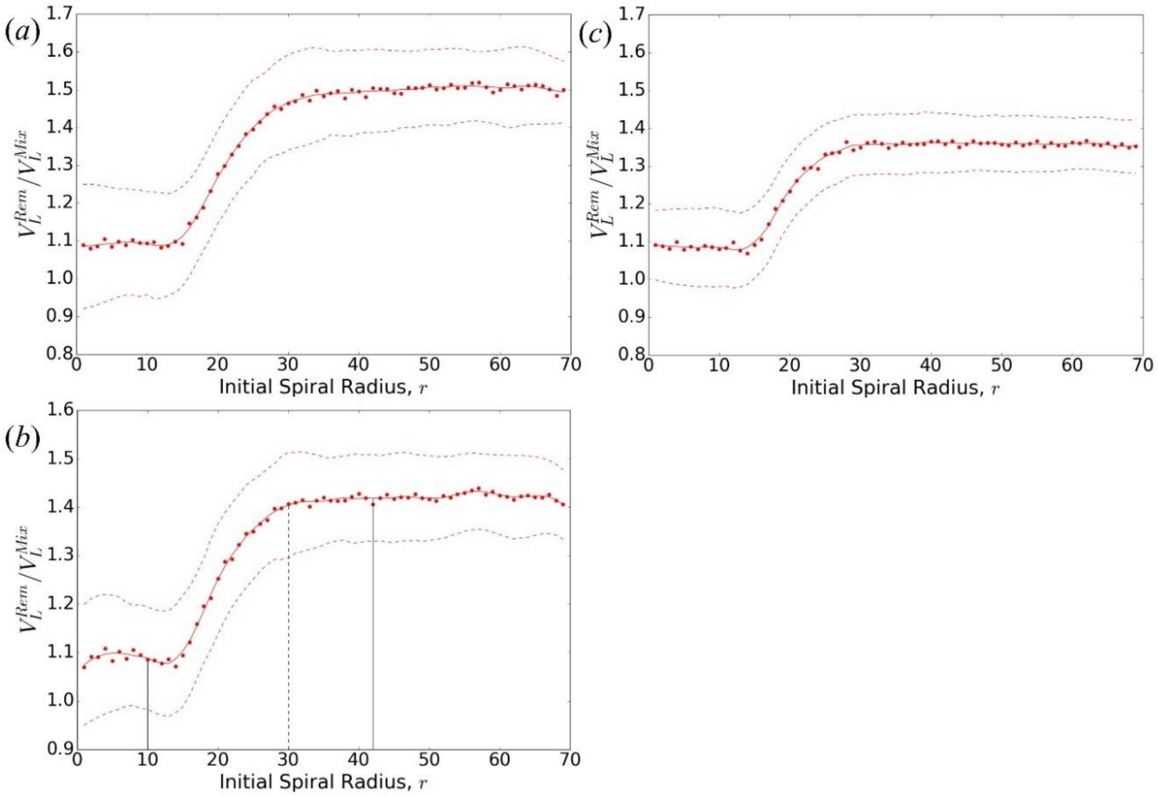

Figure S10. The ratio of the volume fraction of large grains ejected and large grains in the mixture against initial spiral radius, r for spiral digging based on large grains occupying (a) 20%, (b) 25% (a copy of figure 4*c* for comparison) and (c) 30% by volume in the original mixture; V_L^{Rem}: volume fraction of large grains removed from the pit during digging; V_L^{Mix}: volume of large grains in the original mixture.
